# [Retracted] The therapeutic effect of artesunate on rosacea through the inhibition of the JAK/STAT signaling pathway

**DOI:** 10.3892/mmr.2023.13127

**Published:** 2023-11-13

**Authors:** Ting Li, Qingwen Zeng, Xingming Chen, Guojiang Wang, Haiqing Zhang, Aihua Yu, Hairui Wang, Yang Hu

Mol Med Rep 17: 8385–8390, 2018; DOI: 10.3892/mmr.2018.8887

Following the publication of this paper, it was drawn to the Editor's attention by a concerned reader that the GAPDH control western blotting data shown in Fig. 4A were strikingly similar to data appearing in different form in another article written by different authors at different research institutes.

Owing to the fact that the contentious data in the above article had already been published prior to its submission to *Molecular Medicine Reports*, the Editor has decided that this paper should be retracted from the Journal. The authors were asked for an explanation to account for these concerns, but the Editorial Office did not receive a reply. The Editor apologizes to the readership for any inconvenience caused.

